# Total knee arthroplasty with fracture of polyethylene post

**DOI:** 10.1016/j.radcr.2025.06.097

**Published:** 2025-08-01

**Authors:** Bryanna McGowan, Cheng Zhou, Hyunji Shim, Alexandra Aronowitz, Emad Allam

**Affiliations:** Loyola University Medical Center and Loyola Univeristy Chicago, Maywood, IL, USA

**Keywords:** Total knee arthroplasty, Posterior stabilized, Polyethylene fracture, Hardware failure, Post-operative complication

## Abstract

A total knee arthroplasty (TKA) is a common procedure performed in patients with symptomatic osteoarthritis that is refractory to conservative management. The use of polyethylene in prostheses has become the standard in many types of arthroplasties with improved longevity and increased patient satisfaction. We present a case of a rare postoperative complication of polyethylene post fracture detected on CT imaging in a patient who had a primary posterior stabilized total knee arthroplasty (TKA) performed several years prior.

## Introduction

A total knee arthroplasty (TKA) is a common procedure performed in patients with symptomatic osteoarthritis refractory to conservative management with the aim to improve quality of life by relieving pain and enhancing function. However, there is risk of complications despite constant evolution of prostheses materials. The American College of Surgeons National Surgical Quality Improvement Program (ACS NSQIP) has reported that 5.5% of TKA patients experience a major complication requiring medical intervention, 30 days post procedure. Minor complications occur in 2.9% of patients [1].

We present a case of a rare postoperative complication of polyethylene post fracture in a patient with a primary posterior stabilized (PS) TKA that occurred 14 years after the surgery. Practitioners should be aware of this potential complication and the associated radiological findings in patients with primary PS TKA to allow for appropriate and timely management.

## Case presentation

The patient was a 77-year-old male with a history of osteoarthritis status post right PS TKA 16 years ago and left PS TKA 14 years ago presenting with acute left knee pain. The post-operative course had been uneventful. About a week prior to the clinic visit, the patient reported a popping sensation and instability in his left knee while standing up from a kneeling position on the ground. This was followed by pain and the patient now required a cane to ambulate. He denied fevers, chills, erythema, induration, or drainage from the previous surgical site.

Compared to the right knee with TKA prosthesis of the same design, physical examination of the left knee noted more than 5 mm of hypermobility on the anterior/posterior drawer test. There was a notable posterior jump of the tibia with respect to the femur at flexion past 45 degrees. There was a soft endpoint at 90 degrees on the posterior drawer test.

**Fig. 1:**
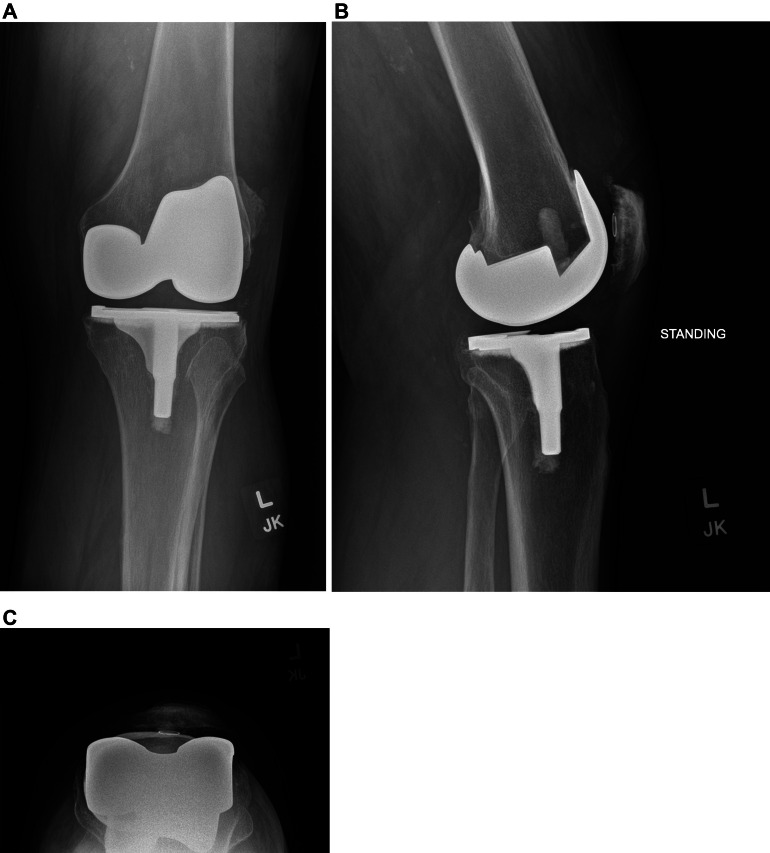
(A), (B), and (C). Frontal, lateral, and tangential patellar radiographs show a left total knee arthroplasty with well-seated components.

**Fig. 2:**
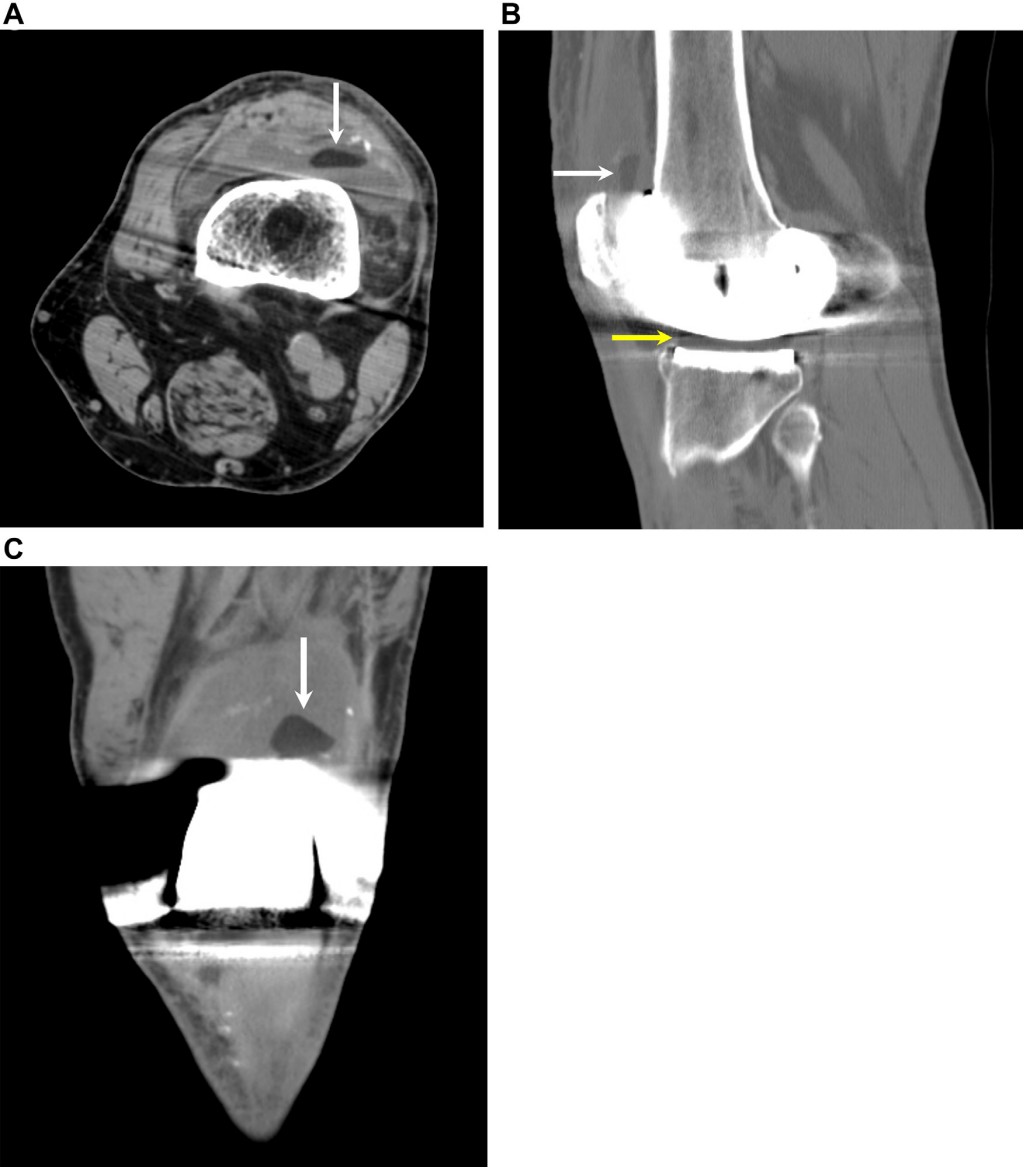
(A), (B), and (C). Axial, sagittal, and coronal CT images show a hypodense structure (white arrow) in the patellofemoral compartment of the left knee joint. The density of this structure is similar to the polyethylene liner (yellow arrow) of the knee arthroplasty.

Radiographs showed no obvious fracture or dislocation (Fig. 1). The metallic components of the prosthesis appeared intact and there was no periprosthetic lucency or osteolysis. However, subsequent CT showed a hypodense structure in the suprapatellar recess of the knee joint (Fig. 2). This was a subtle finding but could be appreciated after adjusting the windows and levels to minimize streak artifact from adjacent hardware. Differential diagnosis based on imaging included a broken post, displaced polyethylene fragment from patellar source, or displaced polyethylene fragment from the tibial tray.

The patient underwent revision left TKA surgery which confirmed a broken and displaced post. Follow up 6 weeks after surgery demonstrated less than 5 mm of mobility on the anterior/posterior drawer test. Imaging showed a well-fixed prosthesis with no signs of loosening.

## Discussion

Although primary TKAs are highly effective, the incidence of revision TKAs has been rising and is projected to grow by 601% between 2005 and 2030 [2]. Polyethylene is the standard material utilized for prostheses. The degradation of polyethylene prostheses due to wear and tear is a major problem that leads to revision of TKAs. Advancements in material engineering have created stronger versions of polyethylene. Antioxidant polyethylene is a prosthetic material that combines an antioxidant compound with the polyethylene. The use of antioxidant polyethylene in primary TKAs is increasing compared to the use of non-antioxidant polyethylene prosthetics [3]. Our patient was reported to have non-antioxidant polyethylene prosthesis for his primary TKA which was subsequently replaced with oxidized zirconium polyethylene prosthesis. When compared to other antioxidants, such as cobalt-chromium alloy, oxidized zirconium has been shown to decrease polyethylene wear by 42% [4].

In addition, the surgical methods of cruciate retaining (CR), medial stabilized (MS), and posterior stabilized (PS) TKA also need to be considered to reduce revision TKAs. Although preferences differ among surgeons, recent data suggests the difference in results between the methods is less than what is considered the minimum clinically important difference (MCID).

A CR TKA retains the posterior cruciate ligament (PCL) while a PS TKA removes the PCL [5]. The PS TKA method is most commonly utilized for TKA, and was also used in our patient for both his primary and revision TKA [6]. A “cam-post” mechanism is utilized in such a design and it provides anteroposterior stability to the backward motion of the femur. However, it also produces an abnormal forward motion of the femur referred to as “paradoxical motion”. This motion contributes to wear of the polyethylene insert/post by increasing the force of the quadriceps femoris. The MS TKA method utilizes a “ball-and-socket” mechanism to minimize wearing of the polyethylene insert and physiologically mimic the movements of natural knee joints. Despite the theoretically improved physiology of the MS TKA mechanistic design, the data remains largely inconclusive whether there is a statistically significant difference between all approaches across multiple analyses.

Polyethylene post fracture is a known complication of PS TKA, but is difficult to diagnose on imaging. On radiographs, the fracture of the polyethylene tibial post is not visible because the polyethylene is radiolucent. However, there may be abnormal alignment of the knee joint, such as anterior or posterior subluxation of the tibia or genu recurvatum [[7], [8], [9]]. On CT, a square or rectangular hypodense intraarticular body may be seen, representing the displaced fragment [10]. Visualization of the fracture is often limited due to beam hardening artifact from the metallic hardware. On MRI, the polyethylene tibial post has low signal on both T1 and T2 weighted sequences. MRI may demonstrate deformity or blunting of the polyethylene post with a square or rectangular hypointense intraarticular body [11].

Polyethylene post fracture has been previously described on arthrogram, CT arthrogram, and MRI, but not on standard CT. This case report underscores awareness of this complication and the subtle findings that may be visible on CT. CT is more convenient to obtain than arthrogram or MRI and may be diagnostic if there is a displaced polyethylene fragment that is not obscured by hardware artifact. MRI with metal artifact reduction sequences may be obtained if there is continued clinical concern.

## Conclusion

This case shows a rare postoperative complication of TKA, specifically polyethylene post fracture in a PS TKA. This may be radiographically occult and cross-sectional imaging is recommended for detection of this complication if clinically suspected. This is not necessarily traumatic and may occur several years after surgery due to polyethylene wear.

## Patient consent

Informed consent for this case was obtained from the patient.
